# Fluorescence optical imaging in pediatric patients with inflammatory and non-inflammatory joint diseases: a comparative study with ultrasonography

**DOI:** 10.1186/s13075-017-1440-8

**Published:** 2017-10-17

**Authors:** Marisa Christin Beck, Anne-Marie Glimm, Sarah Ohrndorf, Kirsten Minden, Ralf Trauzeddel, Stephanie Gabriele Werner, Gerd Horneff, Marina Backhaus, Gerd Rüdiger Burmester, Tilmann Kallinich, Hermann Girschick, Jens Klotsche

**Affiliations:** 1 German Rheumatism Research Center, a Leibniz Institute, Program Area Epidemiology, Charitéplatz 1, 10117 Berlin, Germany; 2 Charité University Medicine Berlin, Rheumatology and Clinical Immunology, Charitéplatz 1, 10117 Berlin, Germany; 3 Department of Pediatric Rheumatology, Helios Hospital Berlin-Buch, Schwanebecker Chaussee 50, 13125 Berlin, Germany; 4 Helios Klinikum Duisburg, Klinik für Rheumatologie, An der Abtei 7-11, 47166 Duisburg, Germany; 5 Centre for Paediatric Rheumatology, Department of Paediatrics, Asklepios Clinic Sankt Augustin, Arnold-Janssen-Straße 29, 53757 Sankt Augustin, Germany; 6 Department of Internal Medicine, Rheumatology and Clinical Immunology, Park-Klinik Weissensee, Schönstraße 80, 13086 Berlin, Germany; 7 Charité University Medicine Berlin, Pediatric Pneumology and Immunology, Augustenburger Platz 1, 13353 Berlin, Germany; 8 Children’s Hospital, Vivantes Hospital im Friedrichshain, Landsberger Allee 49, 10249 Berlin, Germany; 9 Institute for Social Medicine, Epidemiology and Health Economics, Charité University Berlin, Luisenstraße 57, 10117 Berlin, Germany

**Keywords:** Juvenile idiopathic arthritis, Arthralgia, Imaging, Fluorescence optical imaging, Ultrasound, Power Doppler

## Abstract

**Background:**

Valid detection of arthritis is essential in differential diagnosis of joint pain. Indocyanin green (ICG)-enhanced fluorescence optical imaging (FOI) is a new imaging method that visualizes inflammation in wrist and finger joints. Objectives of this study were to compare FOI with ultrasonography (US, by gray-scale (GS) and power Doppler (PD)) and clinical examination (CE) and to estimate the predictive power of FOI for discrimination between inflammatory and non-inflammatory juvenile joint diseases.

**Methods:**

FOI and GSUS/PDUS were performed in both hands of 76 patients with joint pain (53 with juvenile idiopathic arthritis (JIA), 23 with non-inflammatory joint diseases). Inflammation was graded by a semiquantitative score (grades 0–3) for each imaging method. Joints were defined clinically active if swollen or tender with limited range of motion. Sensitivity and specificity of FOI in three phases dependent on ICG enhancement (P1–P3) were analyzed with CE and GSUS/PDUS as reference.

**Results:**

For JIA patients, FOI had an overall sensitivity of 67.3%/72.0% and a specificity of 65.0%/58.8% with GSUS/PDUS as reference; specificity was highest in P3 (GSUS 94.3%/PDUS 91.7%). FOI was more sensitive for detecting clinically active joints than GSUS/PDUS (75.2% vs 57.3%/32.5%). In patients with non-inflammatory joint diseases both FOI and US showed positive (i.e., pathological) findings (25% and 14% of joints). The predictive value for discrimination between inflammatory and non-inflammatory joint diseases was 0.79 for FOI and 0.80/0.85 for GSUS/PDUS.

**Conclusions:**

Dependent on the phase evaluated, FOI had moderate to good agreement with CE and US. Both imaging methods revealed limitations and should be interpreted cautiously. FOI may provide an additional diagnostic method in pediatric rheumatology.

**Trial registration:**

Deutsches Register Klinischer Studien DRKS00012572. Registered 31 July 2017.

## Background

Joint pain is a common complaint in children and adolescents, about 10–20% of school children being affected [1–3]. Distinguishing inflammatory from non-inflammatory causes is an important diagnostic step, but can be challenging. Juvenile idiopathic arthritis (JIA) is the most common chronic inflammatory rheumatic disease in childhood and requires early and adequate anti-inflammatory treatment [4, 5] as it may lead to permanent joint damage and functional disability [6–8]. Non-inflammatory causes of joint pain like idiopathic pain syndromes may require other modes of treatment in an interdisciplinary therapeutic approach [9].

The current International League of Associations for Rheumatology (ILAR) classification criteria of JIA and its treatment strategies and prognosis are, among other criteria, based on the number of inflamed joints involved (<5 vs ≥ 5, oligoarthritis vs polyarthritis) [4, 10], and therefore accurate detection of arthritis in any joint is essential. However, sensitive detection by clinical examination (CE) alone can be difficult, especially in cases of mild arthritis. Ultrasonography (US) in gray-scale (GS) and in power Doppler mode (PD) are established imaging tools for both the detection of synovitis and estimation of current arthritis activity; both techniques have been shown to be more sensitive than CE [11–13]. US allows an immediate, safe, and inexpensive evaluation and is well suited for children. However, it is known to have a relatively high observer dependency [14]. Magnetic resonance imaging (MRI) is regarded as the gold standard for assessment of arthritis in both adults and children [15–18]. However, discrimination between pathological and age-related physiological changes in children and adolescents proves to be problematic [19]. Because of further disadvantages such as high costs, long duration of examination, and the possible need for sedation in younger children, it is not routinely performed in the pediatric rheumatology outpatient clinic.

Indocyanin green (ICG)-enhanced fluorescence optical imaging (FOI) is a novel technology utilizing near-infrared light to visualize altered microcirculation (e.g., neoangiogenesis, hyperperfusion, and capillary leakage) in inflamed joints of the hands and wrists [20, 21]. Advantages of the method are the possibility of examining all joints of both hands (30 joints) together in a relatively short time period (6 minutes), its standardized setting, and the lack of ionizing radiation. However, the application of an intravenous (IV) contrast agent (ICG) might be disadvantageous, although possible side effects are rarely seen.

Comprehensive validation studies on adult patients with different arthritis have shown good agreement of FOI with GSUS, PDUS, and MRI as well as variable sensitivity and specificity dependent on FOI phase and joint, by comparison [22–24]. These results cannot be simply transferred to children and adolescents as the altered vascularity of the juvenile cartilage and growth-related changes might affect the imaging. There are few data regarding its use in pediatric patients, yet first examinations of children by FOI were promising and the examination was well tolerated [25–27].

Our study was designed to determine the association and agreement of FOI with US (GSUS, PDUS) findings and physician assessment of clinical arthritic severity in joints of symptomatic children, as well as to estimate the predictive power of FOI to distinguish between inflammatory juvenile rheumatic diseases and non-inflammatory arthralgia.

## Methods

A total of 76 patients from three pediatric rheumatology centers in Berlin, Germany were recruited for this observational study. The patients were consecutively included. Inclusion criteria were joint pain (due to present JIA or without known reason), age of 6–18 years, and agreement of patient and parents. Exclusion criteria were allergy against ICG or iodine, hyperthyroidism, pregnancy, breast feeding, other known severe diseases, and chronic or active infection of the hands.

The patients were categorized into three groups: group I included 29 JIA patients with clinically relevant active arthritis in the hand region at the time of examination (as determined by pediatric rheumatologist); group II included 23 patients with arthralgia (e.g., caused by hypermobility syndrome, juvenile fibromyalgia, or other arthralgia without inflammatory character); and group III consisted of 53 JIA patients regardless of disease activity and affection of the hand region at the time of examination, and included all patients from group I. All JIA patients included fulfilled the ILAR criteria [10]. In patients with new onset of symptoms and/or uncertain diagnosis at the time of examination, the diagnoses were reconfirmed approximately 1 year later.

The study was performed in compliance with the Declaration of Helsinki. The study protocol was approved by the local ethics committee of the Charité University Medicine Berlin, Germany (No. EA2/126/12). Written informed consent was obtained from all participants.

### Clinical and laboratory examinations

For each patient, a detailed CE was performed in all 71 joints. Joints were defined as clinically active by the presence of either joint swelling or tenderness and limited range of motion. The assessment included a physician’s global disease activity score by a 21-point numerical rating scale (NRS), patient’s/parent’s global assessment of overall well-being by the same 21-point NRS, and an assessment of the patient’s functional capacity by the Childhood Health Assessment Questionnaire (CHAQ) score [28]. The clinical Juvenile Arthritis Disease Activity Score (cJADAS10, range 0–30), which counts any involved joint to a maximum of 10 joints, was calculated for JIA patients [29]. The erythrocyte sedimentation rate (ESR) and C-reactive protein (CRP) were determined for 65 and 71 patients respectively as part of their routine examinations.

### Musculoskeletal ultrasonography

Ultrasonographic examination of both hands was performed in a neutral position by gray-scale (GSUS) and power Doppler (PDUS) ultrasound with the following two machines and settings: Mylab Twice (Esaote, Genova), GSUS 16-MHz frequency, linear probe and PDUS 9.1-MHz frequency, 750-Hz pulse repetition frequency (PRF); and Philips HD15, GSUS 12-MHz frequency, linear probe and PDUS 5.5-MHz frequency, 600-Hz PRF.

The wrist, the interphalangeal joint of the thumb (IP), and the metacarpophalangeal (MCP I–V), proximal (PIP II–V), and distal interphalangeal (DIP II-V) joints were examined and scored semiquantitatively for presence of synovial thickening or joint effusion (combined as synovitis) in GSUS (0 = absent, 1 = mild, 2 = moderate, 3 = marked) and hyperperfusion in PDUS (0 = absent, 1 = presence of up to three single color signals or one confluent signal indicating hyperperfusion, 2 = perfusion signals in less than half of the synovial/joint area, 3 = perfusion signal exceeding half or more than half of the joint) [11].

GSUS and PDUS were analyzed blinded to the clinical characteristics of the patient. For evaluation of interreader agreement between the two pediatric centers conducting sonographic examinations, a random sample survey of 11 static GSUS and PDUS images was taken from patients from center 1 and scored independently by the center 2 pediatric rheumatologist.

### Fluorescence optical imaging

**Fig. 1:**
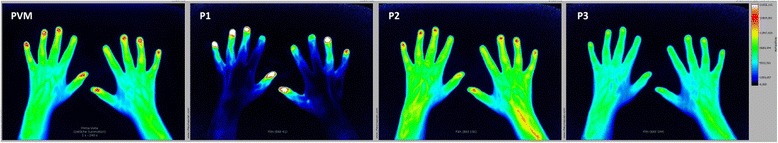
FOI findings in a patient with arthralgia (group II) without signs of inflammation. P1 describes the period between starting the examination and increased signal intensities in the fingertips, the time point of increased signal intensity in the fingertips is analyzed for evaluation. P2 is defined as the phase with persisting high signal intensities in the fingertips, the time point of a red-colored signal in the fingertips is analyzed for evaluation. P3 begins with the absence of signal intensity in the fingertips, and the time point of a missing (no) signal in the fingertips is analyzed for evaluation. *PVM* automatically generated Prima Vista Mode, *P1–P3* phases 1–3

FOI was performed in each patient with the Xiralite® X4 system (Mivenion, Berlin, Germany) following a standardized procedure [22]. Standard examination time was 6 minutes, recording one image per second and hence adding up to a sequence of 360 images. A bolus of ICG with a dose of 0.1 mg/kg body weight was injected as an IV dye 10 seconds after the beginning of image acquisition. For interpretation, both film modus and an automatically generated composite image (Prima Vista Mode (PVM)) were considered. Evaluation was carried out according to Werner et al. [22, 23], who defined three phases of the film modus (P1, P2, P3) based on signal intensity in the fingertips. P1 describes the time between starting the examination and presentation of increased signal intensities in the fingertips, P2 is defined as the phase with persisting high signal intensities in the fingertips, and P3 begins with the absence of signal intensity in the fingertips and lasts until the end of examination (see Fig. 1). Each joint was scored semiquantitatively by color intensity, size, and shape of enhancement from grades 0 to 3 (0 = no signal enhancement, 1 = low signal enhancement (≤25% of affected joint area), 2 = moderate signal enhancement (>25 to ≤ 50% of affected joint area), 3 = strong signal (≥50% of affected joint area)). For each patient the fluorescence optical imaging activity score (FOIAS) was calculated as a sum score over all joints. All FOI findings were analyzed in consensus by two readers who were blinded to the clinical characteristics of the patient (A-MG, MCB).

### Statistical analyses

Data management and statistical analysis were performed using SAS 9.3 (SAS Institute, Cary, NC, USA). Statistical analyses were performed on the patient and individual joint levels. The predictive value for discrimination between patients with inflammatory and non-inflammatory joint diseases by CE, GSUS, PDUS, and FOI was evaluated by calculating the area under receiver operating characteristics curve (AUC). The correlation of CE, GSUS, PDUS, and FOI scores was estimated by the standardized regression coefficient from univariable linear regression analyses. The standardized regression coefficient is reported as a measure of association and can be interpreted as the correlation. A robust variance estimator was used to account for the possible correlation of CE, GSUS, PDUS, FOI, and CE within the study sites [30]. A sum score of the gradings across the 30 hand joints of a patient was considered for the analyses on the patient level. Likewise, the association of the different methods was analyzed on the joint level. Agreement rates were determined at the individual joint level taking into consideration all evaluated joints. In each imaging method, a joint was considered to be affected if its grading was greater than or equal to one (grade ≥ 1) and otherwise to be unaffected (grade 0, normal). CE, GSUS, and PDUS were used as reference methods for determining the absolute agreement, sensitivity, and specificity between various pairs of modalities. In order to investigate possible growth-related changes of FOI during the pubertal growth spurt (i.e., increased vascularization leading to more positive FOI results), we further divided groups I and II into patients aged < 13 and patients aged ≥ 13 and analyzed the respective distribution of the FOI sum scores. Besides, interreader agreement was evaluated by the kappa statistics. *p* < 0.05 was considered statistically significant.

## Results

### Group I

#### Study population

Twenty-nine JIA patients with clinically relevant active arthritis in the hand region were included in this group. Mean age at examination was 13.7 years (standard deviation (SD) = 3.3, median = 14.0); mean number of joints with clinically active arthritis in the hand region was 7.1 (SD = 5.2). Clinical parameters at study enrolment are presented in detail in Table 1.

**Table 1:** Demographic and clinical data of the study population

	Group I: JIA patients with clinically active arthritis in the hand region		Group II: non-inflammatory joint diseases		Group III: all JIA patients	
	(*n* = 29)		(*n* = 23)		(*n* = 53)	
	*n*	%	*n*	%	*n*	%
Gender						
Female	23	79	19	83	45	85
Male	6	21	4	17	8	15
Age (years), mean (SD); median	13.7 (3.3); 14.0		13.8 (2.4); 14.9		13.7 (3.3); 14.1	
Disease duration (years), mean (SD); median	3.3 (4.0); 1.6		1.7 (1.8); 1.1		3.7 (4.1); 2.1	
Diagnosis						
Juvenile idiopathic arthritis	29	100.0	n.a.	n.a.	53	100.0
Systemic arthritis	0	0.0	n.a.	n.a.	1	2
Oligoarthritis, persistent	0	0.0	n.a.	n.a.	6	11
Oligoarthritis, extended	0	0.0	n.a.	n.a.	1	2
Polyarthritis, seronegative	19	66	n.a.	n.a.	28	53
Polyarthritis, seropositive	5	17	n.a.	n.a.	7	13
Enthesitis-related arthritis	2	7	n.a.	n.a.	6	11
Psoriatic arthritis	2	7	n.a.	n.a.	3	6
Other arthritis	1	3	n.a.	n.a.	1	2
Arthralgia	n.a.	n.a.	23	100	n.a.	n.a.
Physician’s global assessment (NRS 0–10)						
Mean (SD)	3.4 (1.9)		n.a.		2.4 (1.9)	
Inactive disease (NRS < 1)	0	0.0	n.a	n.a.	9	17
Therapy						
Nonsteroidal anti-inflammatory drugs	20	69	10	43.5	31	59
Systemic glucocorticoids, low dose	5	17	0	0.0	6	11
Systemic glucocorticoids, high dose	3	10	0	0.0	3	6
Disease-modifying antirheumatic drugs	19	65	0	0.0	32	60
Methotrexate	15	52	0	0.0	23	43
Sulfasalazine	2	7	0	0.0	3	6
Leflunomide	1	3	0	0.0	1	2
Etanercept	4	14	0	0.0	6	11
Adalimumab	0	0.0	0	0.0	3	6
Abatacept	1	3	0	0.0	1	2
Tocilizumab	2	7	0	0.0	2	4
Canakinumab	1	3	0	0.0	1	2
ESR (mm/h), mean (SD); *n*	17.0 (19.2); 24		9.5 (6.5); 21		14.8 (16.1); 44	
CRP (mg/dl), mean (SD); *n*	0.4 (0.5); 28		0.1 (0.1); 23		0.3 (0.5); 48	
cJADAS-10						
Mean (SD)	13.3 (4.8)		n.a		9.5 (5.9)	
Inactive disease (cJADAS-10 ≤ 1)	0	0.0	n.a.		3	6
CHAQ						
Mean (SD)	0.7 (0.6)		0.5 (0.6)		0.5 (0.6)	
No functional disability (CHAQ = 0)	7	24	8	27	16	30
Patient’s Global Assessment (NRS 0–10), mean (SD)	2.6 (2.2)		3.9 (3.4)		2.6 (2.2)	

*JIA* juvenile idiopathic arthritis, *SD* standard deviation, *n.a.* not applicable, *NRS* numerical rating scale, *ESR* erythrocyte sedimentation rate, *CRP* C-reactive protein, *cJADAS* clinical Juvenile Arthritis Disease Activity Score, *CHAQ* Childhood Health Assessment Questionnaire

#### Comparison of CE, US, and FOI

**Fig. 2:**
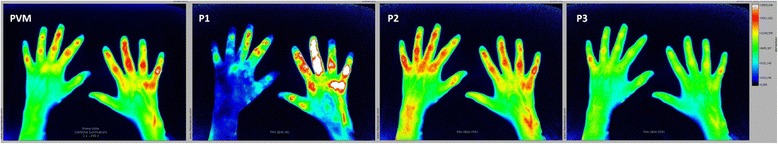
FOI findings in a 17-year-old patient with clinically active seronegative polyarthritis (group I). Increased signal intensities as a sign of active inflammation (synovitis) can be seen in both hands especially in P1 as follows: high signal intensities (FOIAS grade 3) in MCP 4 + 5, PIP 3 + 5, and DIP 3 of the right hand; moderate signal intensities (FOIAS grade 2) in MCP 2, PIP 2 + 4, and DIP 2 of the right hand; and FOIAS grade 1 in IP of the right hand and DIP 2 + 3 and PIP 2 + 3 + 5 of the left hand. *PVM* Prima Vista Mode, *P1–P3* FOI phases 1–3

**Fig. 3:**
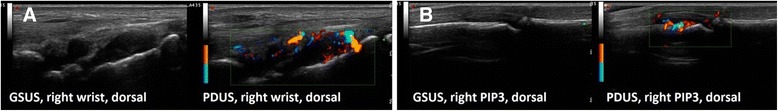
Gray-scale (GSUS) and power Doppler (PDUS) ultrasonography findings in a 13-year-old patient with clinically active seronegative polyarthritis (group I). **a** GSUS synovitis grade 3 and PDUS activity grade 2 as a sign of active synovitis in the right wrist (radiocarpal and intercarpal joint). **b** GSUS synovitis grade 2 and PDUS activity grade 3 (≥50% of the intraarticular area) as a sign of active synovitis in the right PIP3 joint from dorsal. *PIP* proximal interphalangeal joint

FOI findings were compared with US findings (Figs. 2 and 3) and clinical findings in 870 wrist and finger joints.

Prevalence of joints with pathological findings was 206 (24%), 281 (32%), 118 (14%), and 395 (45%) by CE, GSUS, PDUS, and FOI, respectively. Most frequently affected joints in each method were the wrists as well as PIP joints. In FOI, the largest number of increased signal intensities was found in P2 (34% of joints). The number of joints with grading ≥ 2 was 87 (10%) in GSUS, 27 (3%) in PDUS, and 233 (26.8%) in FOI.

**Fig. 4:**
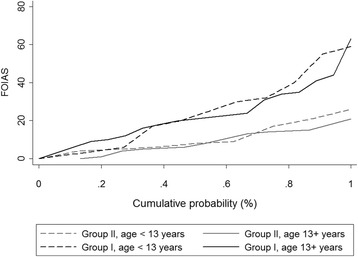
Comparison of cumulative probability plot of fluorescence optical imaging scores (FOIAS) for patients aged < 13 years and patients aged ≥ 13 years in group I (JIA patients with clinically active arthritis in hand region) and group II (patients with non-inflammatory joint diseases)

The age-related distribution of FOI scores showed no relevant differences between patients aged < 13 and patients aged ≥ 13 years (Fig. 4).

#### Sensitivity and specificity

Of the 206 joints rated as clinically active, 57.3%/32.5% were also positive by GSUS/PDUS and 75.2% by FOI. Of the 664 non-inflamed joints by CE, 75.5%/92.3% was negative in GSUS/PDUS and 63.9% was negative in FOI. With CE as reference, FOI sensitivity was highest in P2 (62.6%) and specificity was highest in P3 (93.1%).

Taking GSUS as reference, CE had a sensitivity of 42.0% and a specificity of 85.1% and FOI had a sensitivity of 67.3% and a specificity of 65.0%. With PDUS as reference, CE had a sensitivity of 56.8% and a specificity of 81.5% while FOI had a sensitivity of 72.0% and a specificity of 58.8%. Specificity of FOI compared to US was high in PVM and P3 (GSUS 86.9% and 94.3%; PDUS 84.8% and 91.7%). However, corresponding sensitivities were low (GSUS 28.1% and 18.6%; PDUS 35.6% and 20.3%). Sensitivity of FOI was highest in P1 (GSUS 51.8%; PDUS 59.8%) (Table 2).

**Table 2:** Group I and II: agreement (%), sensitivity (%), and specificity (%) of FOI, CE, GSUS, and PDUS vs CE, GSUS, and PDUS as standards of reference

	CE			GSUS			PDUS		
	Agreement	Sensitivity	Specificity	Agreement	Sensitivity	Specificity	Agreement	Sensitivity	Specificity
Group I: JIA with clinically active arthritis in the hand region									
FOI any phase	66.6	75.2	63.9	65.7	67.3	65.0	60.6	72.0	58.8
PVM	78.2	41.7	89.5	67.9	28.1	86.9	78.2	35.6	84.8
P1	72.3	56.3	77.2	70.5	51.8	79.7	72.1	59.8	74.0
P2	71.8	62.6	74.7	66.7	51.2	74.0	67.9	57.6	69.5
P3	75.3	19.4	93.1	69.5	18.6	94.3	81.9	20.3	91.7
CE	–	–	–	71.1	42.0	85.1	78.2	56.8	81.5
GSUS	71.1	57.3	75.5	–	–	–	80.3	96.6	77.8
PDUS	78.2	32.5	92.3	80.3	40.6	99.3	–	–	–
Group II: non-inflammatory joint diseases									
FOI any phase	75.0	0.0	75.0	72.1	39.4	77.3	75.5	61.5	75.7
PVM	93.3	0.0	93.3	84.6	18.1	95.1	92.3	23.1	93.6
P1	88.0	0.0	88.0	80.1	21.3	89.4	86.6	15.4	88.0
P2	83.2	0.0	83.2	77.1	27.7	84.9	83.3	53.8	83.9
P3	98.2	0.0	98.2	86.0	4.5	98.6	97.1	23.1	98.6
CE	–	–	–	86.4	0.0	100.0	98.1	0.0	100.0
GSUS	86.4	0.0	86.4	–	–	–	87.7	84.6	87.7
PDUS	98.1	0.0	98.1	87.7	11.7	99.7	–	–	–

*FOI* fluorescence optical imaging, *CE* clinical examination (joints with clinically active arthritis), *GSUS* ultrasonography in gray-scale mode, *PDUS* ultrasonography in power Doppler mode, *JIA* juvenile idiopathic arthritis, *PVM* Prima Vista Mode, *P1–P3* FOI phases 1–3

### Agreement rates

Agreement of CE and US (GSUS and/or PDUS) was 71.6%. Agreement of CE and FOI ranged from 71.8 to 78.2% depending on the individual phases of FOI, agreement of GSUS and FOI ranged from 66.7 to 70.5%, and agreement of PDUS and FOI ranged from 67.9 to 81.9%. Highest agreement was found for P3 where mostly negative results were present, the lowest was found for P2 (Table 2). Lack of accordance was primarily due to a higher proportion of positive findings by FOI; in 16.8% of joints only FOI was rated positive. US and FOI consistently detected abnormal findings in 47.6% of clinically active joints and in 14.2% of clinically inactive joints.

### Group II

#### Study population

Twenty-three patients with arthralgia without any known inflammatory rheumatic disease were included in this group. None showed signs of clinical inflammation in any joint region. Mean age at examination was 13.8 years (SD = 2.4, median = 14.9); mean number of joints with arthralgia in the hand region was 5.2 (SD = 7.9). Clinical parameters are presented in detail in Table 1.

#### Comparison of CE, US, and FOI

Figure 1 shows a typical FOI image sequence of a patient with arthralgia but without any increased signal intensity as a sign of inflammation in any phase.

Of the 690 wrists and finger joints evaluated, none had clinical signs of arthritis. However, 17 patients showed abnormalities in GSUS and/or PDUS suggestive of inflammatory activity in 96 joints (14%) (GSUS 94 joints, PDUS 13 joints), and 21 patients showed increased signal intensities in FOI in 172 joints (25%). The number of joints with grading ≥ 2 was 17 (2.4%) in GSUS, one (0.1%) in PDUS, and 53 (7.7%) in FOI. In FOI, P2 showed most increased signal intensities (16.8%), whereas P3 showed least signals and thus was most specific (98.2%). In US, most positive results were found in the wrists and MCP joints, and in FOI most positive results were found in the wrists and PIP joints.

Again, the age-related distribution of FOI scores showed no relevant differences between patients aged < 13 and patients aged ≥ 13 years (Fig. 4).

### Sensitivity, specificity, and agreement rates of US and FOI

Agreement of US and FOI was high, given the fact that most results were expected to be negative. With both GSUS and PDUS as reference, FOI had specificity up to 98.6% (in P3, respectively). However, overall sensitivity was relatively low (GSUS 39.4%; PDUS 61.5%). In 5.5% of joints both imaging methods showed positive findings.

### Groups I and II: predictive value of FOI, GSUS/PDUS, and diagnosis and their correlations

The predictive value for discrimination between inflammatory and non-inflammatory rheumatic diseases was calculated. The area under the curve (95% CI) was 0.85 (0.75–0.95) for PDUS, 0.80 (0.68–0.92) for GSUS, and 0.79 (0.67–0.91) for any FOI phase (P2 = 0.77 (0.64–0.89)).

On the patient level, correlation of US with joints with clinically active arthritis was strong (GSUS, β = 0.63 (*p* < 0.001); PDUS, β = 0.69 (*p* < 0.001)), whereas correlation of FOI with clinically active arthritis was moderate (β = 0.32–0.54 (*p* < 0.001)). Correlation of FOI with US was moderate. P1 was found to correlate most strongly with GSUS (β = 0.50 (*p* < 0.001) and P2 with PDUS (β = 0.51 (*p* < 0.001)) (Table 3). On the joint level, correlation of US with clinically active joints was moderate (GSUS, β = 0.37 (*p* < 0.001); PDUS, β = 0.35 (*p* > 0.001)), as well as correlation of FOI with clinically active joints (up to β = 0.40 (*p* < 0.001) for any FOI phase). Correlation of FOI with GSUS was weak to moderate (up to β = 0.35 (*p* < 0.001) in P2); there was no correlation between FOI and PDUS on the joint level (Table 4).

**Table 3:** Group I and II: correlation of FOI, GSUS/PDUS, and CE on the patient level; standardized β (p value)

	Number of joints with active arthritis	GSUS	PDUS
FOI any phase	0.54 (<0.001)	0.48 (<0.001)	0.43 (0.001)
PVM	0.45 (0.001)	0.38 (0.006)	0.43 (0.001)
P1	0.52 (<0.001)	0.50 (<0.001)	0.38 (0.006)
P2	0.54 (<0.001)	0.45 (0.001)	0.51 (<0.001)
P3	0.32 (0.020)	0.46 (0.001)	0.43 (0.002)
GSUS	0.63 (<0.001)	–	–
PDUS	0.69 (<0.001)	–	–

*FOI* fluorescence optical imaging, *CE* clinical examination (joints with clinically active arthritis), *GSUS* ultrasonography in gray-scale mode, *PDUS* ultrasonography in power Doppler mode, *PVM* Prima Vista Mode, *P1–P3* FOI phases 1–3

**Table 4:** Group I and II: correlation of FOI, GSUS/PDUS, and CE on the joint level; standardized β (p value)

	Joints with active arthritis	GSUS	PDUS
FOI any phase	0.40 (<0.001)	0.35 (<0.001)	0.29 (0.001)
PVM	0.35 (0.001)	0.23 (0.006)	0.20 (0.001)
P1	0.36 (<0.001)	0.35 (<0.001)	0.27 (0.006)
P2	0.36 (<0.001)	0.30 (0.001)	0.25 (<0.001)
P3	0.23 (0.020)	0.23 (0.001)	0.19 (0.002)
GSUS	0.37 (<0.001)	–	–
PDUS	0.35 (<0.001)	–	–

*FOI* fluorescence optical imaging, *CE* clinical examination (joints with clinically active arthritis), *GSUS* ultrasonography in gray-scale mode, *PDUS* ultrasonography in power Doppler mode, *PVM* Prima Vista Mode, *P1–P3* FOI phases 1–3

### Group III

#### Study population

This group consisted of 53 JIA patients with variable current disease activity in the hand region, and included all 29 patients of group 1. Mean age at examination was 13.7 years (SD = 3.3, median = 14.1); mean number of joints with arthritis in the hand region was 4.1 (SD = 5.1). Clinical parameters are presented in detail in Table 1.

#### Findings in CE, US, and FOI

Of 1590 joints evaluated, 218 (13.7%) were classified as clinically active. US was positive in 26% and FOI in 41.3% of all joints examined.

#### Correlations of FOI and US with clinical and functional parameter of disease activity

Synovitis scores and FOIAS were compared with the cJADAS10, physician’s global assessment of disease activity, functional disability scores (CHAQ), and laboratory parameters (ESR, CRP) (Table 5). Correlation between GSUS/PDUS and cJADAS10 was found to be relevant (β = 0.62 (*p* < 0.001)/β = 0.66 (*p* < 0.001)), as well as correlation with physician’s global assessment of disease activity (β = 0.51 (*p* < 0.001)/β = 0.55 (*p* < 0.001)). There was a weak correlation between GSUS/PDUS and CHAQ (β = 0.32 (*p* = 0.018)/β = 0.36 (*p* = 0.008)) and no correlation with the patient-reported outcome measurements pain and fatigue. GSUS/PDUS and inflammatory blood parameters did not show any correlation.

**Table 5:** Group III: correlation of FOI, GSUS, and PDUS with clinical parameters; β (*p* value)

	GSUS	PDUS	FOI any phase	PVM	P1	P2	P3
Physician’s assessment	0.51 (<0.001)	0.55 (<0.001)	0.37 (0.007)	0.37 (0.006)	0.36 (0.008)	0.38 (0.005)	0.29 (0.034)
Number of joints with arthritis	0.59 (<0.001)	0.63 (<0.001)	0.39 (0.004)	0.36 (0.008)	0.36 (0.008)	0.46 (<0.001)	0.30 (0.028)
cJADAS-10	0.62 (<0.001)	0.66 (<0.001)	0.36 (0.008)	0.36 (0.008)	0.34 (0.013)	0.42 (0.002)	0.37 (0.007)
CHAQ score	0.32 (0.019)	0.36 (0.008)	0.10 (0.471)	0.22 (0.106)	0.12 (0.388)	0.21 (0.127)	0.17 (0.238)

*FOI* fluorescence optical imaging, *GSUS* ultrasonography in gray-scale mode, *PDUS* ultrasonography in power Doppler mode, *PVM* Prima Vista Mode, *P1–P3* FOI phases 1–3, *cJADAS* clinical Juvenile Arthritis Disease Activity Score, *CHAQ* Childhood Health Assessment Questionnaire

Correlation between FOIAS and cJADAS10 was moderate (P2, β = 0.42 (*p* = 0.002)), as well as correlations with physician’s global assessment of disease activity (P2, β = 0.38 (*p* = 0.005)). FOI PVM correlated moderately with CRP level (β = 0.48 (*p* = 0.0001)). There was no correlation between FOIAS and CHAQ or between FOIAS and patient-reported outcome measurements of pain and fatigue.

### Interreader agreement for ultrasonography

Interreader agreement was investigated in 11 static GSUS and PDUS images between two blinded independent raters. Interreader agreement was found to be good for GSUS (agreement in 93% of joints, κ = 0.76) and substantial for PDUS (agreement in 100% of joints, κ = 1.00), when considering the number of joints with any positive signal. The agreement rate for the grading was slightly lower for GSUS (agreement in 60% of joints, κ = 0.61) and PDUS (agreement in 66% of joints, κ = 0.70). The highest proportion of disagreement was found between grading 1 and 2 (GSUS, 20% of joints; PDUS, 26% of joints).

### Safety

In all subjects, the procedure was well tolerated. One patient presented circulatory problems due to peripheral IV insertion before the fluorescent compound was applied. The FOI examination could be performed after recovery. No adverse events were observed.

## Discussion

ICG-enhanced FOI with the Xiralite® system is a novel imaging technique that has been shown to visualize inflammation in arthritis of wrist and finger joints and has been evaluated in various validation studies in adult rheumatology [22–24, 31, 32]. The aim of this study was to acquire data regarding the use of FOI in children and adolescents with joint diseases by comparing it to findings in both sonographic and CE.

### Patients with juvenile idiopathic arthritis

Consistent with previous studies in adults [22–24], FOI showed a higher rate of positive findings than the other compared modalities. Most signals were found in P2, which was also the most sensitive phase compared to CE (62.6%). This supports the hypothesis that it might be the most valuable phase for detecting subclinical activity [22–24]. However, there is a need for further evaluation to prove this.

Agreement of FOI and CE was comparable to agreement of GSUS/PDUS and CE, whereas FOI overall sensitivity for detecting clinically active arthritis was higher than GSUS/PDUS sensitivity (75.2% vs 57.3%/32.5%).

Agreement of FOI and US was good, especially agreement with PDUS (up to 81.9%), which reflects the fact that both FOI and PDUS display acute inflammatory changes rather than chronic changes. Sensitivity of FOI compared to US was moderate. The highest values were reached if increased signal intensities in any phase were considered (GSUS 67.3%, PDUS 72.0%). Analyzing the phases separately, highest sensitivity was found for FOI P1 compared to hyperperfusion in PDUS in JIA patients with clinically active arthritis (59.8%) with corresponding high specificity (74.0%). This supports the theory by Werner et al*.* [22] that P1 reflects high synovial vascularization and thus correlates best with disease activity [33]. However, these results distinguish from results of previous studies comparing FOI to US which found P2 to be the most sensitive phase [22–24].

In accordance with adult studies, highest specificity compared to both GSUS and PDUS was found for P3 (94.3% and 91.7%). This was the phase showing least signal intensity increase; presumably it reflects increased capillary permeability with abnormal persistency of ICG and is therefore mostly found positive in osteoarthritis [22, 23, 34].

Differences in the characteristics of the FOI phases compared to previous studies might result from the higher variation of ICG distribution patterns we observed in children and adolescents compared to adults. Defining the phases according to the standardized protocol was difficult in several cases, where the ICG distribution deviated from the known characteristic flow behavior. This is possibly due to growth-related vascular changes and might have had an influence on sensitivity and agreement rates of the individual phases. Therefore, the current standard of interpretation in adult rheumatology by Werner et al. [22, 23]—in particular the definitions of the phases—might have to be reevaluated for pediatric rheumatology. This could also be concluded by means of the moderate correlations of US and FOI scores on the joint and patient levels (Tables 3 and 4). We found no relevant differences in the distribution of FOIAS scores between patients aged < 13 years and patients aged ≥ 13 years (see Fig. 4). Nevertheless, further age-related examinations are needed for evaluation of growth-related changes in FOI.

Both FOI and US detected a high number of positive results in clinically asymptomatic joints of JIA patients. In adult rheumatology, it is known that residual synovitis in patients with rheumatoid arthritis in clinical remission is frequent and predicts the risk of relapse and ongoing structural joint damage [35]. Even though there are follow-up reports suggesting that subclinical arthritis detected by US also predicts development of clinical arthritis in JIA patients [36], direct evidence from comparing US to histopathological findings—as has been provided for adults—has not been (and most likely will not be) tested for children. Pre/post comparisons of adult patients with inflammatory arthritis in clinical remission suggested that positive signals in FOI in clinically asymptomatic joints may also predict a relapse after premature withdrawal of treatment [37]. A recent study found FOI to be particularly sensitive in detecting clinically silent inflammation in joints that were positive by US [31].

In our study, agreement of US and FOI in clinically inactive joints that presented abnormalities in imaging was relatively low (up to 25.4%), showing that such preclinical/subclinical findings in children and adolescents have to be interpreted with caution. After all, validity of US in pediatric rheumatology has still not been fully established and preliminary standardized definitions of synovitis in US were established only recently [38]. Additionally, it is known that there is a wide range of growth-related variations in joints of children and adolescents that can be seen on imaging. In MRI studies of patients with JIA, changes in bone shape, signal intensity, and the amount of joint fluid were found that were partly unrelated to disease activity [39]. Moreover, bony depressions resembling erosions are frequently seen on MRI of healthy children [19]. Therefore, the clinical importance of such findings in both MRI and FOI remains to be determined and it is unclear whether FOI findings in asymptomatic joints of JIA patients demonstrate subclinical inflammation and thus predict development of clinically apparent arthritis. In the future, follow-up studies could help in evaluating their significance.

### Patients with arthralgia without any known inflammatory rheumatic disease

This group served as a control group, because none of the patients presented clinical signs of an inflammatory joint disease. Previous examinations of healthy controls and individuals with arthralgia without any sign of inflammatory rheumatic disease showed a low rate of positive findings in FOI (0–5%) [22, 23]. However, a vast majority of 91% of this group had increased signal enhancements in at least one joint and phase. Also, 74% of the patients showed abnormalities in US suggestive of synovitis.

Remarkably, there was only moderate agreement between the positive results detected by US and FOI, suggesting that the mechanisms causing such findings differ between the two techniques. This is most likely due to the fact that FOI is based on different physical principles than US, such as light optics and microangiography. Thus, it should be considered a complementary rather than a competitive imaging method.

The high number of positive signals in both US and FOI resulted from many joints being scored grade 1. Excluding those joints greatly minimized positive results and led to high specificities (FOI 94.5–99.2%, US 97.4%). For clinical use, this could mean that FOI tends to overestimate findings and that discreet findings with scores < 2, especially in P2, should be interpreted with caution in children with suspected inflammatory rheumatic diseases, as they might not be a sign of active arthritis but of mechanical stress or blood flow alterations. As discussed before, this possibility should also be considered for patients with inflammatory diseases with positive signals in clinically inactive joints. However, any finding in P3 should be taken seriously, as this highly specific phase might reflect the presence of inflammation.

Most positive results in both US and FOI in patients with non-inflammatory diseases were found in the wrists. Interestingly, these results are compatible with observations in a healthy control group whose wrists were examined by MRI, where a high prevalence of increased volumes of joint fluid, signal changes similar to bone marrow edema, and bony depressions resembling erosions were found [19]. Both MRI and FOI findings might reflect mechanical stress through the high use of this part of the hand during daily activity.

The predictive value for discrimination between active inflammatory and non-inflammatory conditions was calculated. We found it to be comparably high for both GSUS/PDUS and FOI (0.80/0.85 and 0.79) with slight advantages for US, showing that they are equally valid methods for differential diagnosis in children and adolescents with unclear joint pain.

### Limitations

Our study has some limitations. Specific limitations of the technology include the lack of visualization of anatomic structures as well as the limited ability to assess palmar inflammation due to overlying structures reducing the depth of light penetration, most notably in the area of MCP joints. Also, FOI is currently available for examination of hands and wrists only, whereas the most common category of JIA—oligoarthritis—is often manifest in the knee or ankle joint. Therefore, the method might be more valuable for polyarticular-course JIA. Furthermore, it is limited to older children as the method’s fixed setup requires the patient’s capability to keep still for 6 minutes as well as a minimum arm’s length. The youngest patient examined in our study was 6.5 years old.

FOI demonstrates any inflammation of the hand region including scratches or psoriatic plaques, which can result in signal enhancement similar to synovitis. Even though experienced readers are able to identify signals caused by such skin lesions, any clinical findings should be documented in order to be considered in image interpretation [40].

FOI is a procedure that includes an IV injection with potential side effects, like circulatory problems and allergic reactions. In our study, FOI examination was tolerated well, with one report of circulatory problems due to peripheral IV insertion before the fluorescent compound was applied.

The study population was rather inhomogeneous including several patients with unclear clinical findings at the time of examination. In hindsight, this limitation could be reduced by verifying every patient’s diagnosis approximately 1 year after the examination. Another limitation was the use of two different US machines with potential variance in resolution and display.

Ultimately, we were confronted with the problem that there is no assured gold standard for detection of synovitis in children, which is why sensitivities and specificities should be regarded with reserve. Because of the fact that MRI is an invasive technique and not a routine procedure in pediatric rheumatology, we were not able to include it as a reference method in our study.

## Conclusions

ICG-enhanced FOI is a new imaging technique in rheumatology. It detects and excludes inflammatory changes in joints of children and adolescents in good agreement with sonography and CE.

FOI had the highest rate of positive findings of all methods and showed signal enhancements in a majority of patients without known inflammatory rheumatic diseases, which is why discreet findings should always be put in a clinical context. Sonographic examination also showed limitations, proving that despite its value in clinical routine it cannot be regarded as an assured standard of reference in pediatric rheumatology. After all, both diagnostic methods need to be interpreted with caution in the use of children in order not to overestimate their findings. Diagnosis of patients with unclear clinical findings remains challenging. However, FOI is a simple, non-ionizing, and well-tolerated method that could serve as an additional diagnostic tool in pediatric rheumatology. In order to further evaluate the value of this method, and particularly its subclinical findings, more studies in pediatric rheumatology are needed.

## Abbreviations

AUC: Area under receiver operating characteristics curve
CE: Clinical examination
CHAQ: Childhood Health Assessment Questionnaire
cJADAS: Clinical Juvenile Arthritis Disease Activity Score
CRP: C-reactive protein
DIP: Distal interphalangeal
ESR: Erythrocyte sedimentation rate
FOI: Fluorescence optical imaging
FOIAS: Fluorescence optical imaging activity score
GS: Gray-scale mode
ICG: Indocyanin green
ILAR: International League of Associations for Rheumatology
IP: Interphalangeal
IV: Intravenous
JIA: Juvenile idiopathic arthritis
MCP: Metacarpophalangeal
MRI: Magnetic resonance imaging
NRS: Numerical rating scale
P: Phase
PD: Power Doppler mode
PIP: Proximal interphalangeal
PMV: Prima Vista Mode
PRF: Pulse repetition frequency
SD: Standard deviation
US: Ultrasonography
